# Verbal Comprehension Ability in Aphasia: Demographic and Lexical Knowledge Effects

**DOI:** 10.1155/2014/258303

**Published:** 2014-01-23

**Authors:** Panagiotis G. Simos, Dimitrios Kasselimis, Constantin Potagas, Ioannis Evdokimidis

**Affiliations:** ^1^School of Medicine, University of Crete, Voutes Campus, 71003 Heraklion, Greece; ^2^Department of Neurology, University of Athens Medical School, Greece

## Abstract

*Background*. Assessment of sentence-level auditory comprehension can be performed with a variety of tests varying in response requirements. A brief and easy to administer measure, not requiring an overt verbal or a complex motor response, is essential in any test battery for aphasia. *Objective*. The present study examines the clinical utility of receptive language indices for individuals with aphasia based on the Comprehension of Instructions in Greek (CIG), a variant of the Token Test, and the Greek version of PPVT-R. *Methods*. Normative data from a large community sample of Greek adults aged 46–80 years was available on both measures. A word-level-independent measure of auditory comprehension was computed as the standard score difference between the two tests and used to compare patients with and without comprehension deficits as indicated by their Boston Diagnostic Aphasia Examination profile. *Results and Conclusions*. Indices of internal consistency and test-retest reliability were very good. Education and age effects on performance were significant, with the former being stronger. The potential clinical utility of differential ability indices (contrasting sentence- and word-level auditory comprehension tests) is discussed.

## 1. Introduction

Auditory comprehension is one of the major components of general linguistic ability and many individuals with aphasia demonstrate comprehension deficits. These deficits are commonly associated with lesions in various left hemisphere regions, including the posterior middle temporal gyrus, the anterior superior temporal gyrus, the superior temporal sulcus, the angular gyrus, and frontal areas BA 46 and BA 47 [1]. In general, auditory comprehension is assessed at two levels: at the word level (word level auditory comprehension, henceforth WLAC) and at the sentence level (sentence level auditory comprehension, henceforth SLAC). In clinical settings, comprehension of spoken sentences is considered more critical and predictive of overall linguistic and social functioning and requires a set of intact cognitive functions, including lexical/semantic access (primarily tapped by WLAC tasks), syntactic processing, and working memory [2–4].

The ability to extract meaning from spoken sentences can be assessed with a variety of task formats. Some tests were specifically designed to assess processing of particular syntactic sentence frames, such as the Sentence Comprehension Test of the Northwestern Assessment of Verbs and Sentences [5, 6], the Subject-relative, Object-relative, Active, Passive syntactic battery [7], and the Syntactic Comprehension test included in the Bilingual Aphasia Battery ([8] also available in Greek: [9, 10]). The majority of tests employed in routine clinical practice, however, were designed to provide a global measure of comprehension of spoken language. Such tests include the Complex Ideational Material and Commands subtests of the Boston Diagnostic Aphasia Examination (BDAE; [11] also available in Greek: [12] Papathanasiou, Kasselimis, and Simos, in preparation). Similar tasks are included in the Western Aphasia Battery [13]. Stand-alone tests of SLAC assessing the ability to understand and respond to simple verbal commands, such as the Token Test [14], are also available. Although these tests were not designed to identify deficits in the appreciation of particular syntactic structures, they correlate highly with auditory comprehension and language production scores [2], are rather sensitive to even mild aphasic impairments [2, 15], and, given that they do not require a verbal response, are considered very useful in the evaluation of persons with severe nonfluent aphasia, particularly in the presence of significant time constraints on the assessment procedure.

One key issue involved in the interpretation of deficits documented on single SLAC tests concerns the specificity of results, which may be limited by the concurrent presence of word-level comprehension deficits in some patients. In principle, combining performance on separate scales to assess WLAC and SLAC separately may help improve sensitivity for a more global assessment of auditory comprehension, as well as specificity for detecting sentence-level comprehension difficulties. Such an approach requires normative data on both WLAC and SLAC tests, preferably in the same representative population sample. Given the wealth of evidence demonstrating age and education level effects on both types of tests [12, 16–20], the use of age- and education-adjusted norms is also important in this endeavor. Few studies thus far have contrasted the ability to comprehend verbal instructions with lexical knowledge in order to provide a more informative measure of sentence comprehension, controlling for individual variability in word-level comprehension [21–24].

In the present study, we assessed the psychometric properties (including demographic effects) of a stand-alone SLAC test, consisting of a modified version of the Token Test (originally developed in [14], henceforth referred to as Comprehension of Instructions in Greek-CIG). Further, the psychometric properties and clinical application of a differential measure of SLAC (controlling for WLAC ability) were examined. The Peabody Picture Vocabulary Test-R (PPVT-R, [25]), adapted in Greek by Simos et al. [20], was chosen as measure of WLAC, as it features identical response requirements (manual pointing to target) with CIG. The PPVT-R was designed as a receptive vocabulary test and performance on this test loads primarily on verbal comprehension-related factors [26]. Further, the two tasks pose similar demands for decision making (given that the participant is asked to choose between several alternative stimuli). While acknowledging the obvious limitations of assessing such a complex function, as auditory comprehension, through a single task, we argue that such a test could be a useful neuropsychological tool for assessing SLAC, since it engages most, if not all, of the fundamental processes involved in this function. The clinical sample consisted of 22 individuals with aphasia secondary to stroke who were classified as comprehension-impaired or comprehension-unimpaired based on their BDAE profiles. Data from a large (*N* = 480) community sample of Greek adults were used to compute standard scores on both tests, making it possible to assess group effects on performance differences between CIG and PPVT-R. It was hypothesized that use of a word-level-independent measure of auditory comprehension could improve detection of global comprehension deficits in persons with aphasia and be better equipped to discriminate such difficulties from deficits restricted to sentence comprehension with preserved word comprehension. It was predicted that a sizable percentage of aphasia patients who displayed global comprehension deficits (as assessed by BDAE) would score in the impaired range on CIG despite normal-range performance on word comprehension. Conversely, encountering patients who show the reverse performance profile would be much less likely.

## 2. Methods

### 2.1. Participants 

#### 2.1.1. Community Cohort

Participants were 480 individuals aged 46–83 years recruited from 8 broad geographic areas of mainland Greece. All participants reportedly had normal or corrected to normal vision and hearing and were native Greek speakers. To further ensure that sensory deficits did not affect performance, examiners were trained to observe signs of hearing loss during the preliminary clinical interview. Individuals who appeared to have trouble understanding the examiners' queries at normal, conversational voice level were not included in the data set. Additional exclusion criteria included history of neurological or psychiatric disease or head injury resulting in loss of consciousness >10 min. Test-retest data were obtained from 20 participants within a period of 1-2 weeks. Data collection was performed between 9-2007 and 2-2009. Detailed demographic information on the normative sample has been reported previously by our group [20].

Individuals with aphasia included 22 men aged 33–80 years (mean: 57.82, SD: 10.88 years) with 6–17 years of formal education (mean: 10.45, SD: 4.18 years). All patients reported normal or corrected to normal vision and hearing and were native Greek speakers. All individuals with aphasia were evaluated by a trained neuropsychologist at the Eginition University Hospital. Testing was performed between 4 and 12 months after stroke and included the Greek adaptation of the Boston Diagnostic Aphasia Examination [12]. With the exception of two, all participants were evaluated >6 months after stroke. On the basis of their BDAE scores, standard BDAE profiles were generated, according to which patients were classified into five taxonomic categories: Broca's (*n* = 9), Wernicke's (*n* = 2), global (*n* = 8), transcortical motor (*n* = 2), and transcortical sensory (*n* = 1) aphasia. Patients were then divided into two groups: comprehension unimpaired (CU group consisting of patients with Broca's and transcortical motor aphasia; *n* = 11) and comprehension impaired (CI group consisting of patients with Wernicke's, global, and transcortical sensory aphasia; *n* = 11). The two groups did not differ in age (*P* > .07) or years of formal education (*P* > .85; see Table 1).

### 2.2. Materials

Several versions of the Token test are available [28–34] featuring sets of plastic tokens of various sizes, shapes, and colors. For the purposes of the present study, we adopted the pictorial format of the Token Test introduced by Korkman et al. [35] and included in the NEPSY battery. Initially, 14 verbal instructions were devised to represent increasing levels of difficulty (in verbal complexity and short-term/working memory load). The stimulus consisted of a plate depicting five crosses and four circles varying in color (blue, yellow, red, black, and white) and arranged in a 3 × 3 grid. The participant was asked to point to one or two shapes in a particular sequence specified by the examiner. In pilot testing, all items were administered to 70 men and women aged 50–70 years without history of neurological or psychiatric disorder. Pilot data (item difficulty estimates based on proportion of individuals responding correctly and item-total correlations) did not indicate the need to eliminate any items or change the order of item presentation. All CIG items were administered.

The Greek adaptation of the PPVT-R was used to assess WLAC [20] consisting of 173 stimulus plates. Changes in the target stimulus were deemed necessary on several plates following pilot testing as well as changes in the order of presentation of several items. Cronbach's alpha was .98 and test-retest reliability was estimated at *r* = .88. PPVT-R administration to adult participants started with item 50. The administration was discontinued after 8 errors on 10 consecutive responses. In case of an incorrect response within the first 6 items (items 50–55), reverse administration was implemented until a baseline of 6 consecutive correct responses was reached.

Test administration was conducted individually by trained examiners. Short breaks were taken as required. Participation in the testing was voluntary and participants were informed that they could discontinue at any time.

### 2.3. Analyses

Item-level exploratory analyses on CIG were first performed on the data from the community sample (*N* = 480). The stability index for the total score was satisfactory (test-retest *r* = .70). Chronbach's alpha was .76 (all item-total correlation coefficients were >.3 with the exception of item no. 1 which was associated with near perfect performance and close to zero variance in this sample). Zero-order and partial correlation coefficients were used to estimate the effects of demographic variables and divide the sample into age- and education-level subgroups. Further, linear multiple regression analyses were implemented in order to ensure that age and education did not, independently, exert significant influence on CIG scores within each subgroup. Next, the effect of gender as well as the interaction between age and education on CIG raw scores was assessed through an ANOVA with gender age group, and education level group as the between-subjects variables. Finally, demographically adjusted standard scores were computed for PPVT-R and CIG, as well as difference scores reflecting differential ability on SLAC and WLAC. Two sets of difference scores were computed: simple algebraic difference and using Payne and Jones' [27] method which takes into account the intercorrelation between the original test scores in the normative population using the formula *Z*_*D*_ = *Z*_CIG_ − *Z*_PPVT_/√2 − 2*r*_*xy*_.

Finally, the utility of each set of standard scores for identifying subtle SLAC deficits in the absence of word-level comprehension difficulties was examined in the patient data. This aim was pursued at both the group level and for individual patients. At the group level, we assessed the magnitude of aphasia subgroup differences on the three metrics (CIG, PPVT, and CIG-PPVT difference scores). We also cross-tabulated aphasia subgroup against the proportions of patients demonstrating impaired performance on PPVT alone, CIG alone, and on both tests (as indicated by scores falling below the 5th percentile in the respective normative distributions).

## 3. Results

### 3.1. Normative Data

Correlations of raw CIG scores were moderate with both age (*r* = −.36, *P* < .0001, partial correlation controlling for years of education: *r* = −.29) and years of formal education (*r* = .43, *P* < .0001, controlling for age: *r* = .37). Accordingly, correction for education was deemed necessary in order to obtain standard scores, which were especially important for evaluating performance differences between CIG and PPVT-R. Demographic correction was achieved by dividing the community sample into 9 age (46–56, 57–67, and 68–83 years) by education subgroups (0–6, 7–12, and 13+ years). With group size >45 in all cases, this breakdown ensured that the independent effect of each of the two demographic variables (controlling for the other variable) was nonsignificant (|*β* | <.3, *P* > .05).

Descriptive information on CIG performance as a function of age and education level is presented in Table 2. The gender (2) by age (3) by education level (3) ANOVA on CIG raw scores revealed significant main effects of the latter two factors (*P* < .001) which were superseded by a significant age by education level interaction, *F*(4,471) = 5.31, *P* = .0001. Follow up tests revealed that the simple main effect of age (indicating decreasing performance with advancing age) was significant for persons with elementary, *F*(2,158) = 8.58, *P* = .0001, *η* ^2^ = .10, or high-school education, *F*(2,142) = 7.98, *P* = .001, *η* ^2^ = .10, and not for participants with tertiary education, *F*(2,173) = 2.68, *P* = .06, *η* ^2^ = .10. In the two former education level groups significant performance differences (Bonferroni-corrected at *a* = .05) were restricted between the youngest and oldest groups. The effect of gender was negligible (*P* > .9).

However, CIG raw score distributions were positively skewed (skewness = −.50, kurtosis = .48, and Kolmogorov-Smirnov index significantly different from 0, *P* < .001). In order to correct this problem, raw scores were first converted to percentiles, separately for each subgroup. These age and education level-adjusted percentile scores were subsequently normalized using Blom's [36] formula. The resulting distributions of scores displayed the features of the normal distribution (skewness and kurtosis ranging between −.3 and +.3 and Kolmogorov-Smirnov indices not significantly different from 0 at the .05 level). For the entire community sample, the mean of the resulting *z* score distribution was −.01 (SD = .94).

Importantly, the association between CIG and PPVT-R scores in the community sample was in the moderate range (zero order *r* = .56; controlling for age and education *r* = .39). Having confirmed that performance on a measure of receptive vocabulary, that does not require an overt verbal response (PPVT-R), contributed significantly to scores on our measure of auditory comprehension at the sentence level, we explored the distribution and potential clinical utility of difference scores between the two tests in the patient sample. Age- and education-level-corrected *z* scores were used in these analyses. PPVT-R scores were normalized separately for the 9 subgroups, according to the results of the corresponding normative study [20]. Difference scores (CIG minus PPVT-R *z* score) were subsequently computed for each participant. These scores were distributed normally (skewness = −.02, kurtosis = .30, and Kolmogorov-Smyrnov = .63, *P* = .8) with a mean of −.03 and a standard deviation of 1.03 and were therefore suitable for estimating critical values indicating extremely poor SLAC in the presence of adequate WLAC. Correlations between each single test and the difference score were in the moderate range (*r* = .56 for CIG and −.58 for PPVT-R).

### 3.2. Patient Data

Table 3 summarizes raw and demographically adjusted scores (PPVT-R, CIG, and CIG-PPVT-R difference scores) for controls, patients with comprehension deficits, and patients without comprehension deficits. Correlations between each single test and the difference score were in the moderate range (*r* = .42 for CIG and −.59 for PPVT-R). Nonparametric Mann-Whitney tests for two independent samples were used to compare the two patient groups, given that the normality assumption was not met (Shapiro-Wilk statistic >.73, *P* < .002). As shown in Table 3, patients with clinically evident comprehension deficits showed lower PPVT-R (raw scores: M-W *U* = 11.0, *z* = −3.10, *P* = .001; demographically adjusted scores: M-W *U* = 20.0, *z* = −2.47, *P* = .014) and CIG-PPVT difference scores (M-W *U* = 22.0, *z* = −2.32, *P* = .02) than patients without such deficits. However, the group difference failed to reach significance for CIG scores (raw or adjusted, *P* > .1). As expected, the two approaches for computing difference scores provided virtually identical results.

Inspection of Table 4 reveals that, among CU patients, only 3/11 showed deficits on CIG and PPVT-R as compared to 8/11 CI patients. Among the remaining CU patients, four scored above the 5th percentile on both tests and four showed significantly reduced performance on CIG in the presence of relatively spared word-level auditory comprehension ability. Conversely, all CI patients scored below the 5th percentile on CIG with three showing relatively unimpaired word-level auditory comprehension ability.

## 4. Discussion

Two key issues which could be addressed through the current data set are discussed in turn below: (a) the dependence of test performance upon demographic factors and (b) the clinical utility of CIG and CIG-PPVT difference scores.

### 4.1. Effects of Demographic Variables

Age effects on SLAC tests (decreasing performance with increasing age) have been reported as minimal (and nonsignificant) for middle- and older-aged individuals [17, 18, 29, 37, 38]. To our knowledge, only one study found significant age effects [39]. However, age effects have been reported for differently designed SLAC tests. For example, Beaumont et al. [16] did find that performance on the Putney Auditory Comprehension Screening Test (PACST) decreased with age. But PACST, although a SLAC test, does not require execution of complex commands but simple yes/no answers. Like age, gender effects are reported as minimal and nonsignificant by many authors [18, 37]. There are also conflicting reports on the effects of years of formal education on SLAC test performance. For instance, Strauss et al. [40] detected minimal educational-level effects on the Token Test with a sample of adults with at least 8 years of education. Other studies, however, have found education-level effects on SLAC test performance [17, 18]. Mansur et al. [19] analyzed the performance of 162 normal subjects on BDAE and found that years of formal education had an effect on WLAC (comprehension of forms, colors, and numbers), while both age and education had an effect on SLAC (Complex Ideational Material).

In the present data set, age effects were relatively small, yet statistically significant, suggesting that SLAC is not resistant to aging in contrast with a number of previous studies [17, 18, 29, 37, 38]. Changes in the functional capacity of the brain with normal aging have been widely reported, and include neuronal loss and generalized atrophy, myelin changes, and the appearance of sporadic neurofibrillary tangles [41–43]. Further, ageing is associated with increased frequency and severity of a host of medical problems, the impact of which on test performance was explored in more detail in a recent report [44]. Additionally, a reduction in everyday level of mental and/or physical activity among older participants (all individuals aged 68–83 years in the current cohort were retirees) should also be considered as a potential contributing factor to age-related decline in test performance [45–47]. It should be noted, however, that age effects were notably weaker for participants with more than 10 years of education. This finding is in accordance with the notion that life-span cognitive changes are moderated by education [48].

Our results agree with previous studies concerning the existence of a substantial effect of formal education on test scores [17, 18]. Indeed, the effects of level of education, indexed by years of formal schooling, outweighed those of age. This result confirms previous studies [17, 18], reporting significant effects of educational level on SLAC test performance. Thus, our data challenge the claim of Strauss et al. [40], who suggested that, for testing subjects who have received at least 8 years of education with the Token Test, a score correction is not needed. Strong effects of education level (and age) have been reported in previous studies on Greek community cohorts on naming [20, 49]. Educational level may affect performance on verbal tests, and especially those measuring lexical knowledge, indirectly as a proxy for higher professional attainment, further formal linguistic experience, cognitive reserve, and, even, as an indicator of higher experience with formal testing situations [50]. In addition, years of education may be considered as a reflection of intrinsic intellectual abilities fostering educational advancement. Some researchers, however, regard educational level with skepticism, suggesting instead the use of performance-based measures of intellectual capacity to adjust test scores [51].

### 4.2. Clinical Utility of CIG and CIG-PPVT Difference Scores

Estimates of internal consistency and test-retest reliability for the entire standardization sample on both tests (i.e., PPVT-R, CIG: [20], and present study) were adequate for clinical use [52]. In this context, both tests could be very useful in clinical practice. First, they are rather brief (administration time does not exceed 7 minutes for CIG and PPVT-R short form) and do not require a verbal or a complex motor response. Therefore, they are suitable for assessing severely nonfluent individuals with aphasia or stroke patients with hemiplegia. Moreover, the partial correlation between PPVT-R and CIG, controlling for age and education, was in the moderate range, suggesting that a significant proportion of individual variance in an SLAC measure can be accounted for by word-level (lexical) knowledge.

Having met minimum psychometric requirements, we then sought to explore the potential clinical utility of differential ability indices across the two tests. Very few studies have thus far directly compared measures of auditory comprehension at the word and sentence level, which do not require an overt verbal response. A common problem when testing individuals with aphasia is that their premorbid verbal IQ is difficult to estimate reliably. Thus, the clinician cannot measure the effect of lexical knowledge on sentence comprehension. By administering two tests (one SLAC test and one WLAC test), both not requiring a verbal response, and by computing the difference between the *z*-scores of these two tests one could, in principle, obtain a more “pure” index of sentence-level comprehension. In the present data set, 11/22 patients were classified as impaired on PPVT-R and 18/22 on CIG, using age- and education-adjusted normative scores. However, only 11/22 patients scored in the impaired range on both tests. This finding is in agreement with the notion that auditory comprehension is not unidimensional, but rather a complex function served by several component processes [1, 53–55]. This notion is consistent with the observation that some patients encounter severe difficulty in appreciating complex syntactic structures (even in tasks that pose minimal demands on single-word lexical/semantic knowledge), while their comprehension of single words is relatively spared. The opposite trend is less common, however.

While the comprehension-impaired group was expected to have low scores on both tests, nonimpaired patients' auditory comprehension abilities are ipso facto considered to be intact. However, this was not the case. Overall, 7/11 patients scored lower than expected on CIG, with 3/11 demonstrating impaired performance also on PPVT-R. This is in accordance with the notion that a clinical, screening battery such as BDAE may not possess adequate sensitivity to reveal the full extent of a given patient's language deficits (see also [49, 56]). It may also be the case, however, that results of a single test, such as CIG, are not sufficient to draw firm conclusions on the integrity of a highly complex language function.

In this context, the present paper argues that the combined use of WLAC and SLAC test scores may provide a comprehensive description of auditory language comprehension disturbance after stroke. At first glance, the results in Table 4 may appear inconclusive. The two groups could not be easily differentiated in terms of CIG performance. This was confirmed by the lack of a significant difference between the two groups on CIG scores (see Table 3). But when performance on PPVT-R was taken into account, the two groups became evidently distinct on the basis of differential performance patterns (see Table 4). It should also be noted that a criterion of low performance on both tasks—thus indicating a “global” auditory comprehension deficit encompassing both lexical/semantic knowledge and syntactic processing—may be of further use to differentiate between the two groups. While the majority of comprehension-impaired patients (8/11) scored in the deficient range on both tasks, this was not the case for the nonimpaired group, where only 3 patients demonstrated comparably low performance. Interestingly, deficient performance on PPVT-R was not observed among patients in the comprehension-nonimpaired group. The three patients in this group who scored below the 5th percentile on CIG may thus be considered as presenting with a pure syntactic deficit, where lexical/semantic knowledge was preserved, but syntactic processing was affected. The fact that the CIG score difference between the two groups failed to reach significance may be explained in terms of syntactic processing difficulties of the nonimpaired group, considering that 9 out of 11 patients were classified as Broca's aphasics (such deficits have been well described in Broca's aphasia; see, for example, [57]). This argument is further supported by the fact that the two groups differed significantly regarding CIG-PPVT difference score (with the nonimpaired group showing greater discrepancy between CIG and PPVT scores). However, no significant difference was found for the corrected difference score.

One final remark should be made with regard to the use of difference scores in this study and neuropsychological research in general. Difference scores derived from simple subtraction may be unreliable. In the present analyses, the use of the correction formulas suggested by Payne and Jones [27] eliminates many psychometric shortcomings, by adjusting for the intercorrelation between the two measures. However, there are still limitations of the formula, because it presumes that the two scores are normally distributed. This latter assumption may lead to overestimation of the abnormality of an individual's difference score. Crawford et al. [58] have created another formula to overcome this issue which, for samples with *N* > 10, as in the present study, is expected to produce comparable results. In any case, the validity of difference scores is an empirical question that should be assessed in practice (for a detailed discussion on the use of difference scores in neuropsychological research, see [59]).

## 5. Conclusion

In conclusion, the present data confirm previous studies reporting significant educational effects on SLAC. Moreover, results contradict previous findings by demonstrating deterioration in SLAC with advancing age. It should also be pointed out that, as with the majority of similar field studies, the present study was based on a sample of convenience composed of volunteers recruited from a variety of sources, a procedure that carries all the potential limitations of nonrandom sampling. However, care was taken to represent major elderly population groups as indicated by geographical distribution, educational level, and current/past occupation. Finally, preliminary patient data support the potential clinical utility of the combined use of CIG and PPVT-R, for identifying patients with pure sentence-level comprehension deficits.

## Figures and Tables

**Table 1:** Demographic characteristics of the community and patient samples.

	Community sample	Comprehension rating^3^	
		Impaired^4^ (*N* = 11)	Nonimpaired (*N* = 11)
Age (years)^1^	62.88 (9.18)	61.45 (12.08)	54.17 (8.57)
	46–83	33–80	38–67
Education (years)	11.22 (4.62)	10.54 (4.63)	10.36 (3.90)
	0–20	6–17	6–16
Gender			
Men	195	11	11
Women	285	0	0
Occupation^2^			
Manual labor	122	3	2
Clerical	176	7	7
Homemaker	64	0	0
Scientific^3^	118	1	2
Geographic area			
Urban	168	9	10
Rural/small town	112	2	1

^1^Mean (SD) and range. ^2^Current occupation or main occupation prior to retirement. ^3^Professional occupation requiring a university degree, such as doctor, architect, pharmacist, and teacher. ^4^Based on BDAE comprehension domain.

**Table 2:** Means (SD) and interquartile range of CIG total score as a function of age and education in the community sample.

	Age (years)		
	46–56	57–67	68–83
Education (years)			
0–6	10.20 (2.39)	9.06 (2.28)	8.01 (2.70)
	9.0–12.0	7.0–11.0	6.0–10.0
7–12	11.43 (2.09)	11.32 (2.37)	9.71 (2.56)
	9.1–13.0	9.0–14.0	8.0–11.5
13+	11.74 (1.89)	11.64 (1.99)	11.60 (1.83)
	11.0–13.0	11.0–13.0	10.0–13.0

**Table 3:** Performance of patients presenting with and without comprehension deficits and controls on the demographically adjusted CIG, PPVT-R, and CIG-PPVT-R difference scores.

		Individuals with aphasia: comprehension rating	
	Controls (*N* = 480)	Impaired (*N* = 11)	Nonimpaired (*N* = 11)
CIG			
Raw (mean, SD)	10.29 (2.70)	0.63 (0.92)	3.81 (4.11)
*z* (mean, SD)	0.07 (0.95)	−1.79 (0.03)	−1.57 (0.34)
Range	−1.83 to 1.43	−1.86 to −1.75	−1.80 to −0.84

PPVT-R			
Raw (mean, SD)	151.03 (21.08)	87.18 (35.53)**	136.36 (18.85)
*z* (mean, SD)	0.06 (0.96)	−1.61 (0.18)	−0.99 (1.00)
Range	−1.75 to 1.63	−1.75 to −1.11	−1.68 to 1.47

Simple algebraic difference			

CIG-PPVT difference score			
*z* (mean, SD)	0.01 (1.10)	−0.17 (0.19)*	−0.57 (0.72)
Range	−3.34 to 2.95	−0.73 to −0.05	−2.31 to 2.07

Payne and Jones' [27] method			

CIG-PPVT difference score			
*z* (mean, SD)	0.013 (1.22)	−0.19 (0.22)	−0.36 (1.17)
Range	−3.70 to 3.26	−0.81 to −0.05	−2.56 to 2.29

Patient group differences: *P = .02, **P = .01.

**Table 4:** Cross-tabulation of the number of patients scoring in the deficient range on CIG and PPVT-R against comprehension-deficit subgroup.

	Comprehension rating			
	Impaired (*n* = 11)		Nonimpaired (*n* = 11)	
	PPVT			
	High	Low	High	Low
CIG				
High	0	0	4	0
Low	3	8	4	3
